# Biomarkers or biotargets? Using competition to lure cancer cells into evolutionary traps

**DOI:** 10.1093/emph/eoad017

**Published:** 2023-05-26

**Authors:** Anuraag Bukkuri, Frederick R Adler

**Affiliations:** Tissue Development and Evolution Research Group, Department of Laboratory Medicine, Lund University, Lund, Sweden; Cancer Biology and Evolution Program and Department of Integrated Mathematical Oncology, Moffitt Cancer Center, Tampa, FL, USA; Department of Mathematics, University of Utah, Salt Lake City, UT, USA; School of Biological Sciences, University of Utah, Salt Lake City, UT, USA; Huntsman Cancer Institute, University of Utah, Salt Lake City, UT, USA

**Keywords:** evolutionary trap, evolutionary game theory, biomarker, cell–cell competition, chemotherapy, targeted therapy, adaptive therapy

## Abstract

**Background and Objectives:**

Cancer biomarkers provide information on the characteristics and extent of cancer progression and help inform clinical decision-making. However, they can also play functional roles in oncogenesis, from enabling metastases and inducing angiogenesis to promoting resistance to chemotherapy. The resulting evolution could bias estimates of cancer progression and lead to suboptimal treatment decisions.

**Methodology:**

We create an evolutionary game theoretic model of cell–cell competition among cancer cells with different levels of biomarker production. We design and simulate therapies on top of this pre-existing game and examine population and biomarker dynamics.

**Results:**

Using total biomarker as a proxy for population size generally underestimates chemotherapy efficacy and overestimates targeted therapy efficacy. If biomarker production promotes resistance and a targeted therapy against the biomarker exists, this dynamic can be used to set an evolutionary trap. After chemotherapy selects for a high biomarker-producing cancer cell population, targeted therapy could be highly effective for cancer extinction. Rather than using the most effective therapy given the cancer’s current biomarker level and population size, it is more effective to ‘overshoot’ and utilize an evolutionary trap when the aim is extinction. Increasing cell–cell competition, as influenced by biomarker levels, can help prime and set these traps.

**Conclusion and Implications:**

Evolution of functional biomarkers amplify the limitations of using total biomarker levels as a measure of tumor size when designing therapeutic protocols. Evolutionarily enlightened therapeutic strategies may be highly effective, assuming a targeted therapy against the biomarker is available.

## 1 INTRODUCTION

The use of biomarkers to guide clinical decision making is ubiquitous in cancer. Defined as ‘any substance, structure, or process that can be measured in the body or its products and influence or predict the incidence of outcome or disease’ [1], biomarkers serve as quantitative tools for diagnosis, evaluation of response to treatment, and monitoring of disease progression [2]. Many biomarkers can be detected in blood circulation: circulating tumor cells, circulating tumor DNA/RNA (ctDNA/ctRNA), miRNA, exosomes [3, 4], and bodily secretions like ORM1 [5], calprotectin and M2 pyruvate kinase [6]). Others, such as BI-RADS, RECIST, iAUGC and ADC [7, 8], can only be assessed with solid biopsy or specialized imaging.

In cancer, we expect biomarkers to be produced disproportionately by cancer cells, making the measured biomarker concentration proportional to the product of the average production per cell and the total number of cells. It is well known that variation among people in the average biomarker production per cell can be large enough to render biomarkers unreliable as predictors of disease status [1, 9]. However, evolution of biomarker production within a patient, has scarcely been investigated. A recent study proposes using biomarkers obtained from blood-based liquid biopsies and circulating leukocytes to glean insight into spatial and temporal heterogeneity in biomarker production [4]. However, although these methods may be able to capture heterogeneity between primary and metastatic tumors within the patient, they cannot capture variance within a single tumor due to the circulatory, non-local nature of the collected specimens. In addition to making biomarkers more difficult to interpret, this variance provides the raw materials for evolution.

Biomarkers are useful clinical tools when they provide information on the state of the underlying disease. But cancer cells do not produce biomarkers for our convenience. Instead, many biomarkers have important functional roles in cancer cells [10], such as influencing proliferation and cell cycle (Let-7 miRNAs [11–13] and miR-15/16 [14]), apoptosis (Let-7 miRNAs [15] and miR-15/16 [16, 17]), motility and metastasis (CA-125 [18–22], miR-15/16/132 [23, 24], circulating tumor cells [25, 26] and PSA [27]), angiogenesis (PSA [27] and exosomal miR-21 [28]), and even chemotherapeutic resistance (CA-125 [29], miR-21 [30, 31] and tumor-derived exosomes [32]). Due to these functional roles, variation in production rates generates an evolutionary game that indirectly shapes the course of therapy. Ignoring *within-tumor* biomarker variance could distort our ability to assess the response of cancer cells to therapy and to choose the appropriate course of action.

In this article, we construct mathematical models to capture the ‘biomarker game’ among cancer cells. To these core models, we add two kinds of therapy: chemotherapy and targeted therapy. We assume that production of biomarker increases a cancer cell’s resistance to chemotherapy [29] but makes it more susceptible to the targeted therapy. This allows us to set an evolutionary trap for cancer cells: We administer one therapy to the initially heterogenous population. This selects for high biomarker-producing cancer cells in the case of chemotherapy or low biomarker-producing cancer cells in the case of targeted therapy. We can then target these cells with the other therapy [33]. In addition to this simple evolutionary trap, we simulate adaptive therapies [34–38], examine population and biomarker dynamics within the tumor and assess whether these therapies function effectively in the face of the information distortion created by evolution of biomarker production. We demonstrate that increased cell–cell competition (as influenced by biomarker levels, e.g. due to the impacts of differences in motility and angiogenic capabilities on resource acquisition) generally increases therapeutic efficacy by leading to a lower population minimum. We find that cancer’s evolvability is a double-edged sword: a high evolvability can contribute to evolutionary rescue but can also set the cancer up for potent evolutionary traps. Overall, the efficacy of these evolutionary trap strategies is robust to the evolvability of the cancer.

## 2 METHODOLOGY

To investigate how functional biomarkers influence cell–cell competition and response to therapy, we use a mathematical modeling approach [39–42]. Our models, grounded in evolutionary game theory [43–49], take into account the ecological (population) and evolutionary (biomarker level) dynamics of a cancer cell population. The ecological component, which follows classic Lotka-Volterra competition equations [50, 51], tracks interacting populations of cells, where cell type *i* has population *C*_*i*_ and biomarker level *v*_*i*_:


$$\begin{array}{l} \frac{dC_{i}}{dt}=r\left(v_{i}\right)C_{i}\left(1-\frac{\underset{j}{\sum }a\left(v_{i},v_{j}\right)C_{j}}{K}\right)-\delta \left(v_{i}\right)C_{i} \end{array}$$ (1)

The carrying capacity, *K*, is the maximum community density, *r* represents the intrinsic growth rate, *a*(*v*_*i*_*,v*_*j*_) captures competition between cells (e.g. for nutrients or space), and *δ* is the natural death rate. We assume that the intrinsic growth rate *r* and the natural death rate *δ* are increasing functions that represent a proliferation advantage and a biomarker production cost, respectively. To capture improvement in cell–cell competition, such as through increased motility, we make the coefficients *a*(*v*_*i*_*,v*_*j*_) an increasing function of *v*_*j*_, scaled to *a*(*v*_*i*_*,v*_*i*_) = 1 for all *v*_*i*_. We use the following functional forms for growth, death and competition:


$$\begin{array}{l} r\left(v\right)=r_{0}{\left(\frac{v}{v_{0}}\right)}^{\beta_{r}} \end{array}$$

$$\begin{array}{l} \delta \left(v\right)=\delta_{0}e^{\beta_{\delta }\left(v-v_{0}\right)} \end{array}$$

$$\begin{array}{l} a\left(v_{i},v_{j}\right)=\frac{a_{max}e^{\beta_{a}\left(v_{i}-v_{j}\right)}}{a_{max}+e^{\beta_{a}\left(v_{i}-v_{j}\right)}-1} \end{array}$$ (2)

In a monomorphic population, the equilibrium population size is


$$\begin{array}{l} C^{*}\left(v\right)=\left(1-\frac{\delta \left(v\right)}{r\left(v\right)}\right)K \end{array}$$ (3)

We assume this takes on a maximum at a positive finite baseline value of *v *= *v*_0_. The increasing competitiveness function *a* favors higher biomarker production. The *evolutionarily stable strategy* (ESS) [52–55] biomarker production will thus be higher than the *group-optimum* value that maximizes the cancer cell population [33, 56, 57].

We include two types of therapy, each assumed to increase the death rate of the cancer cells: (i) general chemotherapy where a higher *v* confers resistance, and (ii) targeted therapy where a higher *v* increases sensitivity (or, conversely, where a lower *v* confers resistance). We describe chemotherapy with a decreasing effectiveness function *E*_*C*_(*v*) and targeted therapy with an increasing effectiveness function *E*_*T*_(*v*):


$$\begin{array}{l} E_{C}\left(v\right)=\zeta_{C}e^{-m_{C}\left(v-v_{0}\right)} \end{array}$$

$$\begin{array}{l} E_{T}\left(v\right)=\zeta_{T}{\left(\frac{v}{v_{0}}\right)}^{m_{T}} \end{array}$$ (4)

The terms *ζ*_*C*_ and *ζ*_*T*_ control the intensity of chemotherapy and targeted therapy respectively, *v*_0_ serves as a baseline biomarker level, and *m*_*C*_ and *m*_*T*_ capture how biomarker levels impact chemotherapy resistance and targeted therapy sensitivity, respectively. As proposed in earlier work [58], these functional forms were created so that chemotherapy is somewhat effective over a large range of biomarker values, whereas targeted therapy is very effective for high biomarker values and not effective at low biomarker values.

The dynamics with therapy can then be modeled with the *G* function or adaptive dynamics framework [53, 59–62] as


$$\begin{array}{l} \frac{dC}{dt}=G\left(v,u,C\right)C \end{array}$$ (5)

where *G* is the fitness generating function that captures the per-capita growth rate of a cell type with production rate *v* in an environment composed of cells with production rate *u* and population size *C*:


$$\begin{array}{l} G\left(v,u,C\right)=r\left(v\right)\left(1-\frac{a\left(v,u\right)C}{K}\right)-\delta \left(v\right)-E_{C}\left(v\right)\tau_{C}\left(t\right)-E_{T}\left(v\right)\tau_{T}\left(t\right) \end{array}$$ (6)

The functions *τ*_*C*_(*t*) and *τ*_*T*_(*t*) are indicators for chemotherapy and targeted therapy treatment timing, respectively, taking on the value 1 when treatment is on and 0 when it is off.

The evolutionary dynamics, given by Fisher’s fundamental theorem of natural selection [63–65], is derived as the product of heritable variation and the slope of the fitness gradient, or


$$\begin{array}{l} \frac{dv}{dt}=k\frac{\partial G}{\partial v}{|}_{v=u} \end{array}$$ (7)

where *k* is a measure of heritable variation (the evolvability of the trait) and $\frac{\partial G}{\partial v}$ is the selection gradient. Using Eq. 6, the selection gradient is


$$\begin{array}{l} \frac{\partial G\left(v,u,C\right)}{\partial v}{|}_{v=u}=\frac{dr}{dv}\left(1-\frac{C}{K}\right)-r\left(v\right)\frac{\partial a}{\partial v}{|}_{v=u}\frac{C}{K}-\frac{d\delta }{dv}-\frac{dE_{C}}{dv}\tau_{C}\left(t\right)-\frac{dE_{T}}{dv}\tau_{T}\left(t\right) \end{array}$$ (8)

This gradient depends on the treatment environment. In this way, we can track the overall population size and mean marker level. We can find the ESS in any constant environment as the value of *v* where *G*(*v,u,C*) takes on its maximum in the first argument at *v* when *v *= *u*. By the ESS maximum principle [53], we find this by setting Eq. 6 and Eq. 8 to 0 and solving. For our parameter values, this always produces a maximum.

In addition to sequential therapy, we investigate two adaptive therapy protocols. The first is based on the total biomarker level, found as *vC*. When *vC < T*, chemotherapy is administered, and when *vC > T*, targeted therapy is given. The second adaptive therapy protocol is based on the mean marker level *v*. As before, when *v < M* chemotherapy is given, whereas if *v > M*, targeted therapy is administered. The baseline parameter values used in our simulations are given in Table 1. These parameter values were chosen to be biologically plausible, numerically convenient for simulation purposes, and to clearly show differences between the different therapeutic regimes. Since we are using an ODE framework, true extinction as in stochastic or difference equations will be impossible. However, since we are concerned with comparing relative effectiveness of therapeutic strategies rather than absolute efficacy, we do not set a threshold population density for extinction.

**Table 1.** Model parameters and values used in simulations

Parameter	Interpretation	Value
K	Carrying capacity	1
$r_{0}$	Baseline growth rate	0.05
$\beta_{r}$	Growth benefit of biomarker production	1
$v_{0}$	Baseline biomarker level	1
$\beta_{\delta }$	Death cost of biomarker production	1
$\delta_{0}$	Baseline death rate	0.02
$\zeta_{C}$	Chemotherapy intensity	0.03
$\zeta_{T}$	Targeted therapy intensity	0.01
$m_{C}$	Chemotherapy resistance due to biomarker production	1
$m_{T}$	Targeted therapy sensitivity due to biomarker production	5
$a_{max}$	Competition coefficient	2
$\beta_{a}$	Competitive benefit of biomarker production	0.5
k	Evolvability	0.01
T	Total marker threshold	Varies
M	Mean marker threshold	Varies

## 3 RESULTS

### 3.1 Evolutionarily stable states

**Figure 1.**
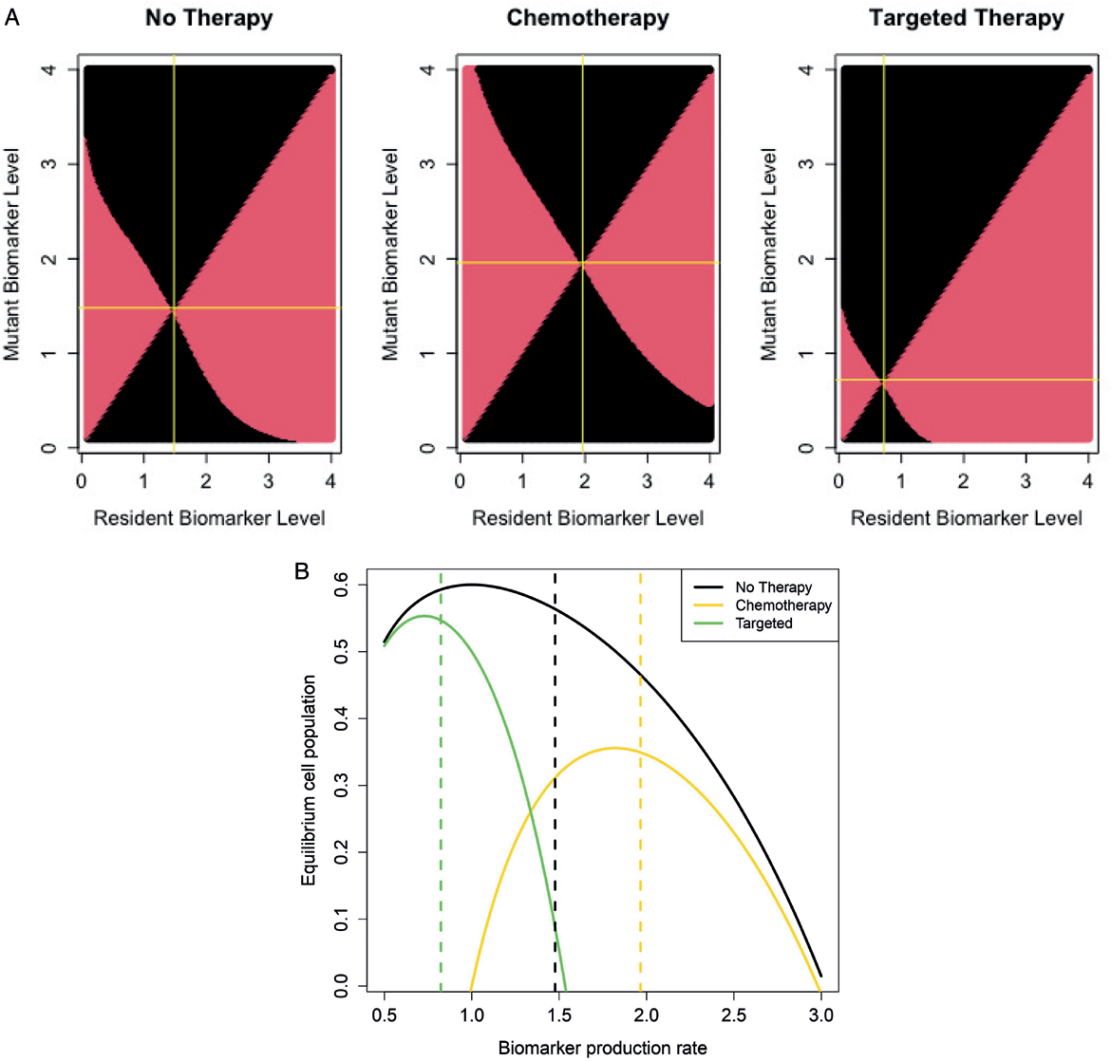
Pairwise invasibility plots and equilibrium population size. In panel A, the horizontal axis represents the biomarker value of the resident type, and the vertical axis the biomarker value of the mutant type. The mutant invades in regions shaded in red and fails to invade in regions shaded in black. The yellow lines show the computed ESS, the resident strategy which no mutant can invade, taking on the values 1.48, 1.96 and 0.72 with no therapy, chemotherapy, and targeted therapy respectively. Panel B shows the equilibrium population size as a function of biomarker production, with the vertical dashed lines the ESS value in the three treatment conditions.

In the absence of therapy, we expect cells to evolve higher production of biomarker than the baseline value *v*_0_ that maximizes equilibrium population size, due to the competitive advantage higher production provides. We call this value the ‘no-therapy ESS’. When chemotherapy is administered, cells will be favored to evolve an even higher level of biomarker production, as this confers resistance to the therapy. We call the resulting ESS the ‘chemotherapy ESS’. Because targeted therapy is more effective for high biomarker-producing cells, we expect it to favor reduced biomarker-producing cells, with a ‘targeted ESS’ less than the no-therapy ESS. To confirm these hypotheses, we create pairwise-invasibility plots in Fig. 1. These plots illustrate the invasion success of rare mutants in a monomorphic resident population [66, 67].

As expected, the chemotherapy ESS is higher than the no therapy ESS and the targeted therapy ESS is lower than baseline (Fig. 1B). Intuitively, this implies that chemotherapy favors higher biomarker production, whereas targeted therapy factors lower production. Furthermore, note where the ESSs (dashed lines) occur relative to the equilibrium population size curves (Fig. 1B). The maximum of the equilibrium population size curves corresponds to a group-optimum: the level of biomarker production that would maximize the cancer cell population size. However, evolution does not operate at the tumor level, but rather at the cellular level. Due to cell competition, an “evolutionary arms race” ensues, resulting in a tragedy of the commons [33, 56, 68, 69], whereby cells increase their biomarker levels, attaining an individual fitness lower than what could be achieved under a team optimum. These findings allow us to set an evolutionary trap, with chemotherapy promoting high biomarker-producing cells and thereby increasing the effectiveness of subsequent targeted therapy and vice versa [33].

### 3.2 Estimation of therapeutic efficacy

**Figure 2.**
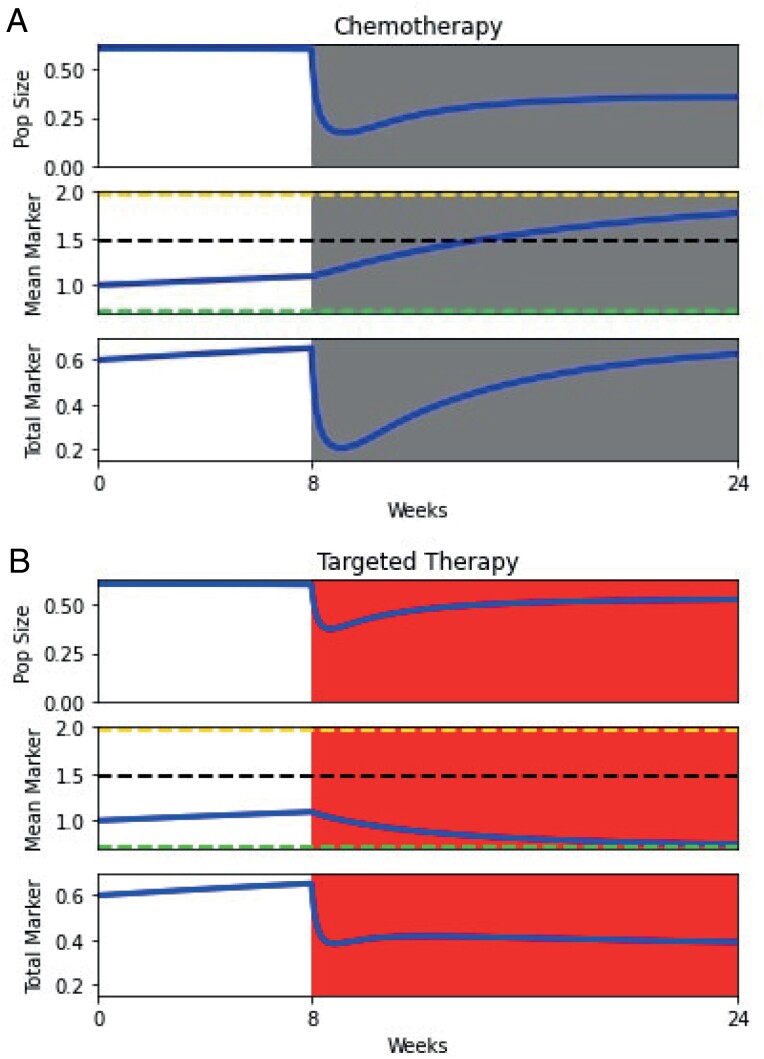
Effects of (A) chemotherapy and (B) targeted therapy on population size, mean marker, and total marker levels. the top panel captures the population dynamics of the cancer cells, the middle panel shows the evolutionary dynamics of mean drug resistance, and the bottom panel shows the total biomarker in the population. Regions shaded in grey and red indicate periods of chemotherapy and targeted therapy. Green, black and yellow dashed lines correspond to the targeted therapy, no therapy and chemotherapy ESSs, respectively. Mean marker levels increase before therapy is administered. The population size (and thereby total marker) initially decreases when therapy is administered, the mean marker increases under chemotherapy, and the mean marker decreases under targeted therapy. Evolution of mean biomarker levels leads to an overestimation of targeted therapy efficacy and an underestimation of chemotherapy efficacy when total biomarker is used as a proxy for population size.

To further examine the effects of chemotherapy and targeted therapy on the dynamics of the population, we simulate a therapeutic period of 16 weeks following no treatment for 8 weeks (pre-diagnosis) in Fig. 2. Although developing targeted therapies against some biomarkers has proven challenging [70], considerable success has been found for others [31, 71–73].

Before therapy is administered, the mean marker level increases because of cell-cell competition. This trend is paralleled in total marker levels. When chemotherapy (targeted therapy) is added, the population size decreases. The selection pressure induced by therapy promotes a rapid evolution of higher (lower) marker levels and evolutionary rescue ensues. The difference between total biomarker and population size dynamics reveals that evolution can alter biomarker production and degrade the value of total biomarker level as a proxy for the size of the tumor. If higher biomarker production increases resistance to chemotherapy, therapeutic efficacy will be underestimated; a physician may conclude that a tumor is progressing despite it having a stable size. Conversely, because targeted therapy favors lower biomarker production, efficacy will be overestimated, potentially leading a physician to conclude that control has been achieved although the tumor has progressed.

**Figure 3.**
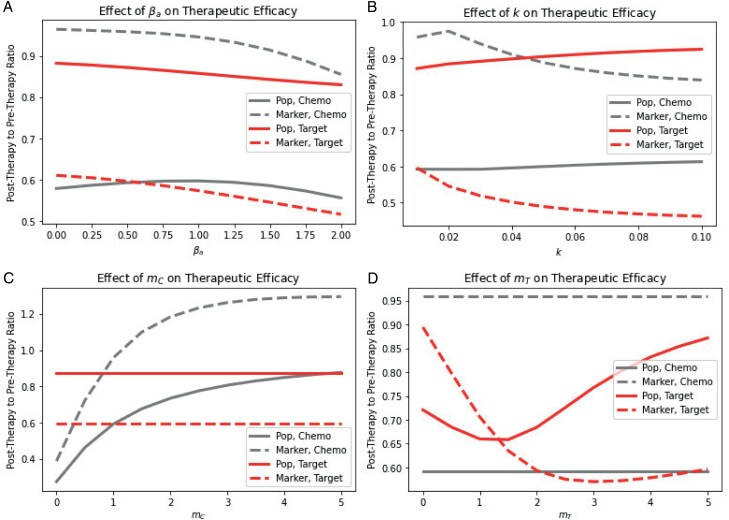
Impact of (A) competition intensity, (B) evolvability, (C) chemotherapy resistance and (D) targeted therapy sensitivity on total biomarker versus population size measures of therapeutic efficacy. For almost all parameter combinations, chemotherapy efficacy is underestimated and targeted therapy efficacy is overestimated.

To quantify these inaccuracies, we compare estimates of therapeutic efficacy if a physician can ascertain the population size of the tumor rather than using the total biomarker proxy. We compare the proportional change in the true population size with the change in total biomarker from the beginning to the end of therapy for chemotherapy and targeted therapy (Fig. 3).

Chemotherapy efficacy is underestimated and targeted therapy efficacy is overestimated for nearly every parameter combination explored. Efficacy is relatively insensitive to the magnitude of competitive intensity and evolvability. Chemotherapy resistance and targeted therapy sensitivity due to biomarker production clearly impact efficacy. Low levels of *m*_*C*_ lead to sharper drops in population size due to chemotherapy, a slower increase in biomarker production, and thereby a slower recovery and lower pre- to post-therapy ratios. Under low targeted therapy efficacy values, the selection pressure induced by competition exceeds that induced by therapy, leading to an increase in biomarker level even when the population is exposed to targeted therapy. This leads to an underestimation of efficacy for targeted therapy, providing the single exception to our observation. Higher levels of *m*_*T*_ lead to more severe drops in population size but also more rapid evolution of lower biomarker levels and recovery in population size. This leads to an overestimation of efficacy. Since we are only concerned with monotherapy here, targeted therapy sensitivity does not impact chemotherapeutic efficacy and vice versa.

### 3.3 Sequential therapy

**Figure 4.**
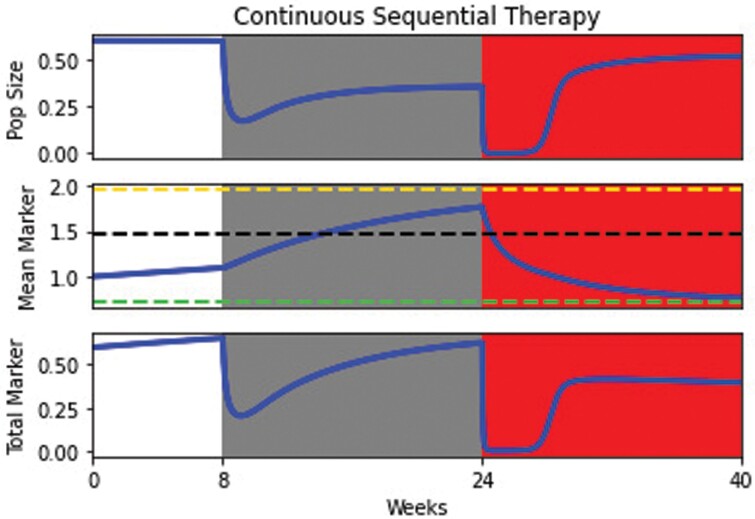
Sequential therapy: the top panel captures the population dynamics of the cancer cells, the middle panel shows the evolutionary dynamics of mean marker levels and the bottom panel shows the total biomarker in the population. Regions shaded in grey and red indicate periods of chemotherapy and targeted therapy. Green, black and yellow dashed lines correspond to the targeted therapy, no therapy and chemotherapy ESSs, respectively. The initial chemotherapy phase reduces the population considerably and selects for higher mean marker levels. This sets up the evolutionary trap: When targeted therapy is administered, the population crashes. If it manages to avoid extinction, the population evolves low mean marker levels.

Since chemotherapy selects for high biomarker-producing cells and targeted therapy selects for low biomarker-producing cells, we hypothesize that an evolutionary trap or double-bind strategy [53, 74–76] in which chemotherapy followed by targeted therapy (or vice versa) could be highly effective for cancer extinction. To implement this, we follow an 8-week period of no treatment (prior to diagnosis) with chemotherapy for 16 weeks and targeted therapy for 16 weeks (Fig. 4). Upon administration of chemotherapy, the population rapidly decreases in size, but evolution of higher biomarker levels provides partial evolutionary rescue. Due to resulting high biomarker levels, administration of targeted therapy nearly drives the population to extinction. If the cancer survives, selective pressure induces evolution toward lower biomarker levels and eventual recovery of the population to near pre-treatment levels.

### 3.4 Adaptive therapy

**Figure 5.**
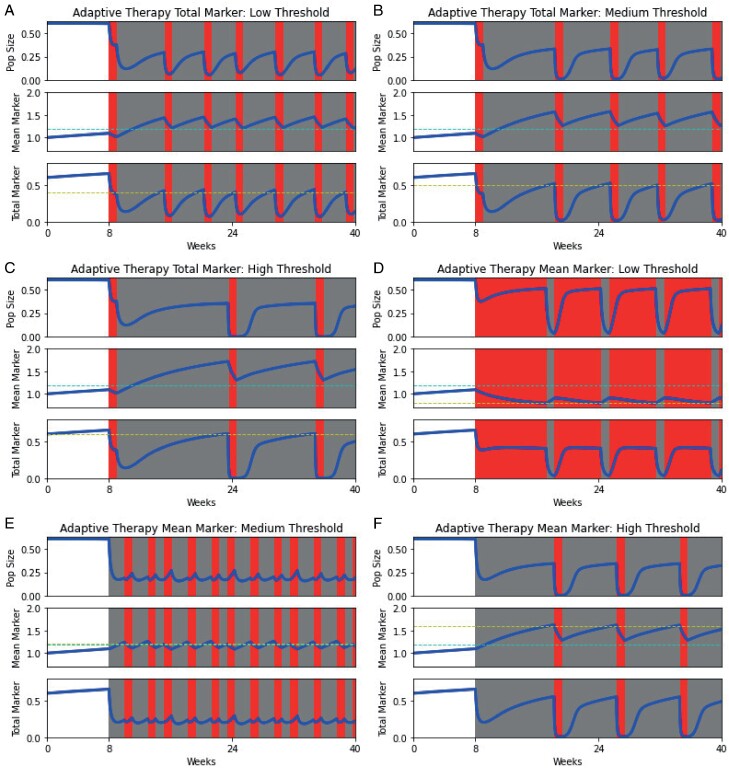
Adaptive therapies based on total and mean marker for low (*T *= 0.4, *M *= 0.8), medium (*T *= 0.5, *M *= 1.2) and high (*T *= 0.6, *M *= 1.6) thresholds. Panels A–C represent adaptive therapy based on total marker, whereas panels D–F represent adaptive therapy based on mean marker. Panels A/D, B/E and C/F represent low, medium and high thresholds for switching therapies. The top, middle and bottom panels in each figure capture the population dynamics, mean biomarker levels and total biomarker levels in the population. Regions shaded in grey and red indicate periods of chemotherapy and targeted therapy. The yellow and cyan dashed lines correspond to the threshold used for switching therapy on and off and the biomarker strategy for which chemotherapy and targeted therapy are equally effective (the chemotherapy-targeted therapy balance). The most effective therapeutic strategies are not those frequently switch between targeted and chemotherapy to use whichever is most effective at any given time. Instead, the most potent strategies result from using thresholds further away from the chemotherapy-targeted therapy balance. This gives enough time for an effective evolutionary trap to be set and drives the population closer to extinction.

To capitalize on the tradeoff between resistance to chemotherapy and sensitivity to targeted therapy, we develop a version of adaptive therapy [35, 37, 38] in which therapeutic choices are based on observed marker levels in the population. We investigate two protocols: one based on total marker, and one based on mean marker level. When only total biomarker data is available (e.g. the level of CA-125 in blood), the first protocol is used: Therapy will be given once the total biomarker surpasses a threshold, *vC > T* (Fig. 5A–C). However, if mean biomarker levels are available (e.g. through single-cell sequencing that detects PSA levels per cell), then the second protocol is used: Therapy will be given once the mean marker exceeds some threshold, *v > M* (Fig. 5D–F). We assume that decisions regarding administration or removal of therapy occur solely at physician visits, here chosen as weekly for convenience, and therapeutic decisions and necessary changes can be made instantaneously. In reality, the frequency of physician visits is highly variable, depending on the type and stage of the cancer as well as more practical logistical constraints. Furthermore, there is often a delay between when a patient’s biomarker levels and measured and when a treatment decision is made. We predict that the optimal threshold should be based on the marker production rate per cell and set to the biomarker level where chemotherapy and targeted therapy are equally effective. If targeted therapy is administered when biomarker levels rise above this threshold and chemotherapy is given when biomarker levels fall below the threshold, cells will always be maximally suppressed.

Panels A–C show adaptive therapy based on total biomarker levels and panels D–F show adaptive therapy based on mean biomarker levels. For both cases, when therapy switches near the chemotherapy-targeted therapy balance (the strategy at which both therapies are equally effective), more frequent switching occurs. As a result, tighter bounds on ecological and evolutionary dynamics are observed. Although these modest fluctuations and relatively stable dynamics may be beneficial for long-term control, it does not allow for an effective evolutionary trap.

**Figure 6.**
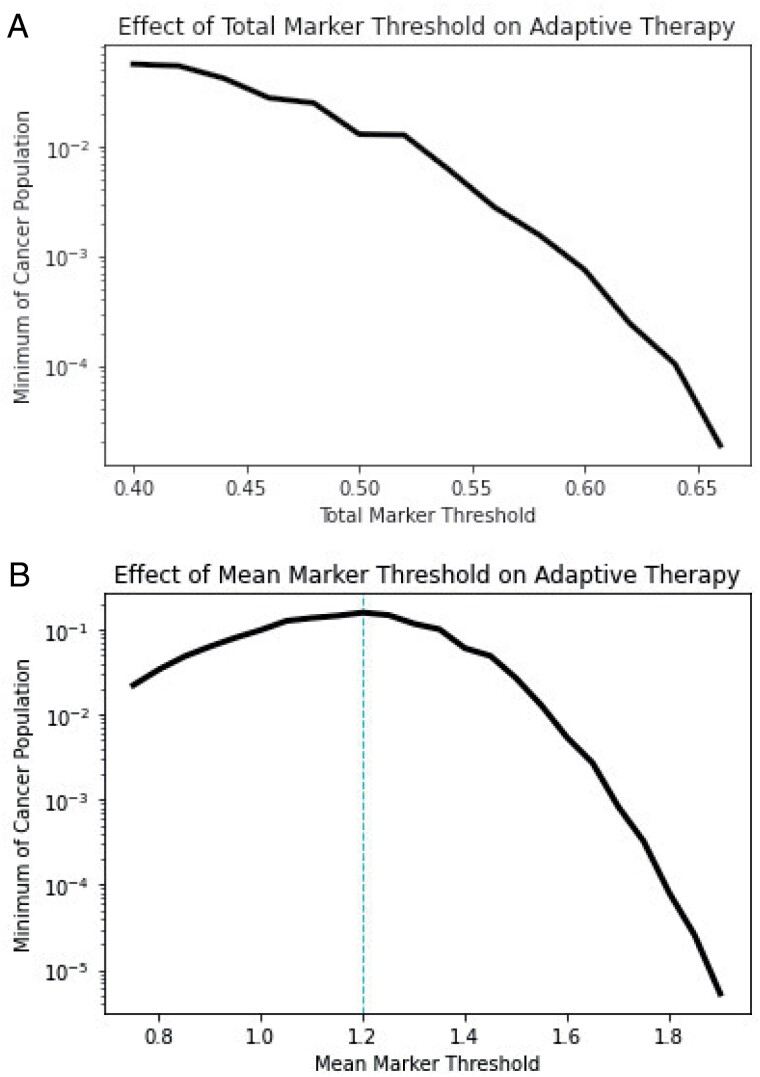
Adaptive therapy experiments: impacts of (A) total and (B) mean marker thresholds. Plotted on log scales. The cyan dashed line corresponds to the biomarker strategy for which chemotherapy and targeted therapy are equally effective (the chemotherapy-targeted therapy balance). Mean marker thresholds further away from the chemotherapy-targeted therapy balance result in lower minima of the population, by allowing for effective evolutionary traps to be set. Similarly, high total marker thresholds give enough time for the population to evolve itself into an evolutionary trap, thereby leading to lower population minima.

When therapy switches further from the chemotherapy-targeted therapy balance, therapies switch less frequently. This allows for more time to set an effective evolutionary trap and results in more drastic fluctuations in population size and biomarker production levels, leading to lower minima of the cancer cell population. To investigate this in more detail, we simulate adaptive therapy protocols for a range of total and mean marker thresholds and plot the minimum of the cancer cell population in Fig. 6. We restrict the range of thresholds to those that result in at least one therapeutic switch. This range is larger when therapy is based on the mean marker than when therapy is based on total marker levels. For the former, this includes both chemotherapy-first protocols (chemotherapy sets up a targeted therapy sucker trap) and targeted therapy-first protocols (targeted therapy sets up a chemotherapy sucker trap). For the latter, this includes only chemotherapy-first protocols. This difference is reflected in the shape of the curves in Fig. 6: Low and high mean marker thresholds correspond to effective evolutionary traps via targeted therapy-first or chemotherapy-first protocols, respectively, whereas levels closer to the chemotherapy-targeted therapy balance correspond to control strategies that are ineffective at extinction with frequent therapeutic switches and tighter ecological and evolutionary bounds. As we can see from Fig. 6B, the further the threshold is set from the chemotherapy-targeted therapy balance, the lower the resulting population minimum. On the other hand, low total marker thresholds correspond to these ineffective extinction strategies (left side of Fig. 6A), whereas high total marker thresholds give enough time for effective traps to be set (right side of Fig. 6A). Contrary to our prediction, the most effective therapeutic strategies for extinction are not those that use the most effective therapy for the current situation but are those that use a currently suboptimal therapy to set up effective evolutionary traps for the future.

### 3.5 Parameter sensitivity

**Figure 7.**
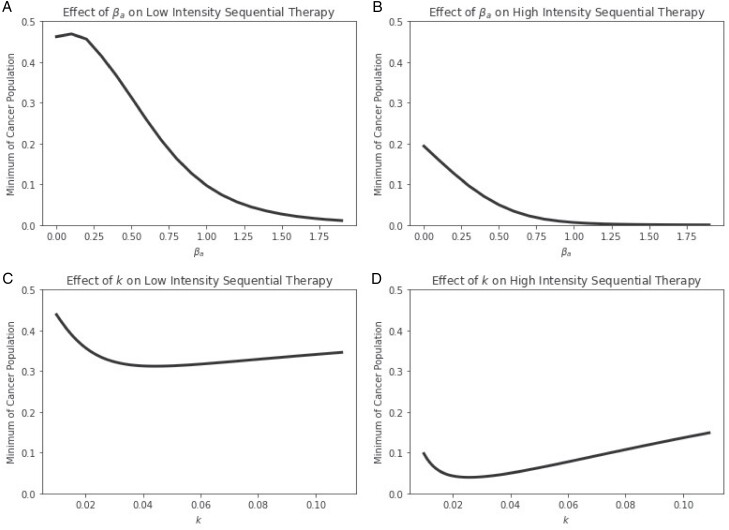
Effects of (A,B) competition intensity and (C,D) evolvability on minimum of cancer cell population for low and high intensity therapy. High competitive benefit pushes the population closer to extinction as it promotes the evolution of higher biomarker levels, setting an effective evolutionary trap for targeted therapy. Evolvability is a double-edged sword: faster evolving species set up more extreme evolutionary traps but are also able to undergo evolutionary rescue more effectively.

Several properties of the cancer and therapy can impact therapeutic efficacy. In this section, we explore how the cancer’s evolvability (*k*), competitive benefit of biomarker production (*β*_*a*_) and therapeutic intensity (*ζ*_*C*_ and *ζ*_*T*_) impact the minimum cancer cell population size under sequential therapy (the evolutionary trap). For high (*ζ*_*C*_ = 0.12 and *ζ*_*T*_ = 0.04) and low (*ζ*_*C*_ = 0.03 and *ζ*_*T*_ = 0.01) intensities of therapy, we simulate ecoevolutionary dynamics using the same sequential therapy protocol as in Section 3.3 and plot the minima of the cancer population for a range of parameter values of *β*_*a*_ and *k* (Fig. 7).

High competitive benefit of the biomarker generally pushes the population to a lower minimum than low competition because it promotes the evolution of higher biomarker levels, setting a more effective evolutionary trap for the targeted therapy. Increasing competition among cells by modifying the microenvironmental landscape [33] could favor higher biomarker production that primes cells for the evolutionary trap. For very low levels of *β*_*a*_, an increase leads to higher minima as it allows the population to evolve higher biomarker levels to buffer the initial chemotherapy onslaught. This trend is more pronounced in the low intensity case than the high intensity case.

Evolvability, the rate at which the cancer generates heritable variation (e.g. through mutation) and thus responds to natural selection, has a double-edged impact. Although cancers with higher evolvability may be lured more quickly into the evolutionary trap, they can also more rapidly evolve the resistance needed to escape. An increase in evolvability at low levels leads to lower minima as the more potent evolutionary trap faced by the more evolvable cancer outweighs the benefits of slightly improved evolutionary rescue. However, as evolvability continues to increase, the tables turn: The benefits of rapid evolutionary rescue overcome the downsides of effective evolutionary traps. These trends are more extreme in the high intensity case, where the selection pressure on cells is strong and evolutionary rescue is critical. Thus, for a given therapeutic dosage, there seems to be an optimal level of evolvability (e.g. minima of Fig. 7C and D) to minimize the nadir of the population based on this trade-off of evolutionary rescue and evolutionary trap efficacy. Generally, higher intensity therapies work relatively better for cancers with low evolvability, whereas low intensity therapies are relatively more effective for cancers with higher evolvability (Fig. 7C and D).

## 4 DISCUSSION

Biomarkers are often used in medicine, and particularly in oncology, to determine the status of underlying disease. However, the information that biomarkers provide may be corrupted by their functional roles in cells [58] and by their consequent evolution. We use mathematical models to investigate the consequences of this complexity, assuming that high biomarker production increases cancer cell growth, competitive ability and resistance to therapy at the cost of a higher death rate, and that we have a targeted therapy that attacks cells with high biomarker levels.

Our modeling study shows that using total biomarker as a measure of population size can lead to incorrect predictions of therapeutic efficacy. Namely, the efficacy of chemotherapy will be underestimated, and the efficacy of targeted therapy will be overestimated (Section 3.2, Figs 2 and 3). We hope that these results inspire physicians to consider the biases that may be introduced when solely using total biomarker as a benchmark for clinical decision-making. Nevertheless, we may be able to take advantage of functional biomarkers to control or even eradicate cancer when chemotherapy and targeted therapy drugs that influence biomarker production are available. Indeed, functional biomarkers have recently been demonstrated to be viable targets for cancer therapy, e.g. CA-125 in ovarian cancer [77, 78]. Chemotherapy can be used to select high biomarker cells, which can then be attacked with targeted therapy. Targeted therapy then favors low biomarker cells that are once more susceptible to chemotherapy (Section 3.3, Fig. 4). We show that these evolutionary traps are more effective at promoting cancer eradication than simply using the most effective therapy at a given moment, even when information about the cancer cell’s population and mean marker levels is available to the physician (Section 3.4, Figs 5 and 6). These traps could be made more effective by promoting biomarker-influenced cell–cell competition. Although these strategies are robust to the evolvability of the cancer, we show that low intensity therapies perform relatively better on highly evolvable cancers whereas high intensity therapies perform better on cancers with low evolvability (Section 3.5, Fig. 7). We hope that our work prompts physicians and researchers to consider evolutionary trap strategies when appropriate drugs are available rather than simply using the most effective therapy at each given time, and devise strategies to improve the efficacy of such traps.

This study has several limitations. First, we make the strong assumption that the biomarker controls a multitude of aspects influencing a cancer cell’s fitness: proliferation, death, resistance and competitive ability. In reality, this assumption is not true for most biomarkers. However, even if biomarker levels only impact cancer resistance (resulting in a classic double bind [53, 74–76]), our notable results surrounding the bias of using solely total biomarker levels and the effectiveness of evolutionary traps still hold. The parameter choices for this theoretical investigation were not estimated from patient data. Future work could calibrate models with time series and single-cell sequencing data. Our evolutionary models are mutation based; if cancer biomarkers change through phenotypic plasticity, responses would be much more rapid and the effectiveness of the therapeutic strategies proposed here would be much less effective [79, 80]. Understanding the mechanisms of biomarker production and the resulting costs and benefits is needed to obtain the accurate eco-evolutionary dynamics that would underlie design and assessment of novel therapeutic strategies.

In addition to the effects on replication, death, competition and resistance considered here, biomarkers play many other functional roles in cancer. For example, PSA activates VEGF-C and VEGF-D, which are involved in angiogenesis and lymphatic metastasis [27]. This creates a public goods game quite different from the competitive models and might require spatial models to capture. Incorporating the role of biomarkers in metastasis, perhaps with lottery, metapopulation or hybrid models, is needed to develop evolutionarily enlightened strategies to control the deadliest cancers.

Medicine has transformed some of the chemicals produced by cancer cells into biomarkers, signals that determine the course of treatment. This transition from accidental by-product of a functional chemical to a signal is ubiquitous in biology. Plants damaged by herbivores create chemicals that have become signals to parasitoids and predators that use them to locate their herbivore hosts [81]. These plants can then manipulate the signal to attract more parasitoids, perhaps even in the absence of herbivore attack. And herbivores can manipulate these signals to reduce attraction of the own enemies [82]. Heterogeneity in signaling among plants creates a signaling game not unlike that of cancer cells. We can think of the body as a plant, herbivores as the cancer, the signals as biomarkers, and the parasitoid as a physician. A judicious combination of general treatment (chemotherapy or pesticide) with something that targets the signal itself might be best at protecting plants and ourselves.

## Data Availability

Codes associated with the plots produced in this paper can be found at https://github.com/abukkuri/BiomarkerGames.

## References

[1] Strimbu K , Tavel JA. What are biomarkers? Curr Opin HIV AIDS 2010;5:463–466. 20978388 10.1097/COH.0b013e32833ed177 PMC3078627

[2] Henry NL , Hayes DF. Cancer biomarkers. Mol Oncol 2012;6:140–6. 22356776 10.1016/j.molonc.2012.01.010 PMC5528374

[3] Marrugo-Ramírez J , Mir M, Samitier J. Blood-based cancer biomarkers in liquid biopsy:` a promising non-invasive alternative to tissue biopsy. Int J Mol Sci 2018;19. 10.3390/ijms19102877 PMC6213360 30248975

[4] Russano M , Napolitano A, Ribelli G et al. Liquid biopsy and tumor heterogeneity in metastatic solid tumors: the potentiality of blood samples. J Exp Clin Cancer Res 2020;39:1–13. 32460897 10.1186/s13046-020-01601-2 PMC7254767

[5] Li F , Yu Z, Chen P et al. The increased excretion of urinary orosomucoid 1 as a useful biomarker for bladder cancer. Am J Cancer Res 2016;6:331–40. 27186407 PMC4859664

[6] Toma SC , Ungureanu BS, Patrascu S et al. Colorectal cancer biomarkers - a new trend in early diagnosis. Curr Health Sci J 2018;44:140–6. 30746161 10.12865/CHSJ.44.02.08 PMC6320460

[7] Dregely I , Prezzi D, Kelly-Morland C et al. Imaging biomarkers in oncology: basics and application to MRI. J Magn Reson Imaging 2018;48:13–26. 29969192 10.1002/jmri.26058 PMC6587121

[8] O’Connor JPB , Aboagye EO, Adams JE et al. Imaging biomarker roadmap for cancer studies. Nat Rev Clin Oncol 2017;14:169–86. 27725679 10.1038/nrclinonc.2016.162 PMC5378302

[9] Aziz N , Detels R, Quint JJ et al. Biological variation of immunological blood biomarkers in healthy individuals and quality goals for biomarker tests. BMC Immunol 2019;20:1–11. 31521107 10.1186/s12865-019-0313-0 PMC6744707

[10] Lin J , Ma L, Zhang D et al. Tumour biomarkers—Tracing the molecular function and clinical implication. Cell Prolif 2019;52:e12589. 30873683 10.1111/cpr.12589 PMC6536410

[11] Akao Y , Nakagawa Y, Naoe T. let-7 MicroRNA functions as a potential growth suppressor in human colon cancer cells. Biol Pharm Bull 2006;29:903–6. 16651716 10.1248/bpb.29.903

[12] Johnson SM , Grosshans H, Shingara J et al. RAS is regulated by the let-7 MicroRNA family. Cell 2005;120:635–47. 15766527 10.1016/j.cell.2005.01.014

[13] Kumar MS , Erkeland SJ, Pester RE et al. Suppression of non-small cell lung tumor development by the ¡i¿let-7¡/i¿ microRNA family. Proc Natl Acad Sci USA 2008;105:3903–8. 18308936 10.1073/pnas.0712321105 PMC2268826

[14] Ofir M , Hacohen D, Ginsberg D. miR-15 and miR-16 are direct transcriptional targets of E2F1 that limit E2F-induced proliferation by targeting cyclin E. Mol Cancer Res 2011;9:440–7. 21454377 10.1158/1541-7786.MCR-10-0344

[15] Tsang WP , Kwok TT. Let-7a microRNA suppresses therapeutics-induced cancer cell death by targeting caspase-3. Apoptosis 2008;13:1215–22. 18758960 10.1007/s10495-008-0256-z

[16] Cai C-kui , Zhao G-yi, Tian L-ying et al. miR-15a and miR-16-1 downregulate CCND1 and induce apoptosis and cell cycle arrest in osteosarcoma. Oncol Rep 2012;28:1764–70. 22922827 10.3892/or.2012.1995

[17] Liang H , Fu Z, Jiang X et al. miR-16 promotes the apoptosis of human cancer cells by targeting FEAT. BMC Cancer 2015;15:448. 26031775 10.1186/s12885-015-1458-8 PMC4450989

[18] Gubbels JAA , Belisle J, Onda M et al. Mesothelin-MUC16 binding is a high affinity, N-glycan dependent interaction that facilitates peritoneal metastasis of ovarian tumors. Mol Cancer 2006;5:1–15. 17067392 10.1186/1476-4598-5-50 PMC1635730

[19] Kaneko O , Gong L, Zhang J et al. A binding domain on mesothelin for CA125/MUC16. J Biol Chem 2009;284:3739–49. 19075018 10.1074/jbc.M806776200 PMC2635045

[20] Rump A , Morikawa Y, Tanaka M et al. Binding of ovarian cancer antigen CA125/MUC61 to mesothelin mediates cell adhesion. J Biol Chem 2004;279:9190–8. 14676194 10.1074/jbc.M312372200

[21] Theriault C , Pinard M, Comamala M et al. MUC16 (CA125) regulates epithelial ovarian can-´ cer cell growth, tumorigenesis and metastasis. Gynecol Oncol 2011;121:434–43. 21421261 10.1016/j.ygyno.2011.02.020

[22] Xiang X , Feng M, Felder M et al. HN125: A novel immunoadhesin targeting MUC16 with potential for cancer therapy. J Cancer 2011;2:280–91. 21611109 10.7150/jca.2.280 PMC3100680

[23] Fabbri M , Paone A, Calore F et al. MicroRNAs bind to Toll-like receptors to induce prometastatic inflammatory response. Proc Natl Acad Sci USA 2012;109. 10.1073/pnas.1209414109 PMC3412003 22753494

[24] Renjie W , Haiqian L. MiR-132, miR-15a and miR-16 synergistically inhibit pituitary tumor cell proliferation, invasion and migration by targeting Sox5. Cancer Lett 2015;356:568–78. 25305447 10.1016/j.canlet.2014.10.003

[25] Aceto N , Bardia A, Miyamoto DT et al. Circulating tumor cell clusters are oligoclonal precursors of breast cancer metastasis. Cell 2014;158:1110–22. 25171411 10.1016/j.cell.2014.07.013 PMC4149753

[26] Chen J-F , Ho H, Lichterman J et al. Subclassification of prostate cancer circulating tumor cells by nuclear size reveals very small nuclear circulating tumor cells in patients with visceral metastases. Cancer 2015;121:3240–51. 25975562 10.1002/cncr.29455 PMC4560974

[27] Jha SK , Rauniyar K, Chronowska E et al. KLK3/PSA and cathepsin D activate˚ VEGF-C and VEGF-D. eLife 2019;8:1–30. 10.7554/eLife.44478 PMC6588350 31099754

[28] Liu Y , Luo F, Wang B et al. STAT3-regulated exosomal miR-21 promotes angiogenesis and is involved in neoplastic processes of transformed human bronchial epithelial cells. Cancer Lett 2016;370:125–35. 26525579 10.1016/j.canlet.2015.10.011

[29] Boivin M , Lane D, Piche A et al. CA125 (MUC16) tumor antigen selectively modulates the sensitivity of ovarian cancer cells to genotoxic drug-induced apoptosis. Gynecol Oncol 2009;115:407–13. 19747716 10.1016/j.ygyno.2009.08.007

[30] Yeung CLA , Co N-N, Tsuruga T et al. Exosomal transfer of stroma-derived miR21 confers paclitaxel resistance in ovarian cancer cells through targeting APAF1. Nat Commun 2016;7:11150. 27021436 10.1038/ncomms11150 PMC4820618

[31] He C , Dong X, Zhai B et al. MiR-21 mediates sorafenib resistance of hepatocellular carcinoma cells by inhibiting autophagy via the PTEN/Akt pathway. Oncotarget 2015;6:28867–81. 26311740 10.18632/oncotarget.4814 PMC4745697

[32] Ciravolo V , Huber V, Ghedini GC et al. Potential role of HER2-overexpressing exosomes in countering trastuzumab-based therapy. J Cell Physiol 2012;227:658–67. 21465472 10.1002/jcp.22773

[33] Bukkuri A , Gatenby RA, Brown JS. GLUT1 production in cancer cells: a tragedy of the commons. NPJ Syst Biol Appl 2022;8:1–13. 35768428 10.1038/s41540-022-00229-6 PMC9243083

[34] Buhler CK , Terry RS, Link KG, Adler FR. Do mechanisms matter? Comparing cancer treatment strategies across mathematical models and outcome objectives. Math Biosci Eng 2021;18:6305–27. 34517535 10.3934/mbe.2021315 PMC10625481

[35] Gatenby RA , Silva AS, Gillies RJ et al. Adaptive therapy. Cancer Res 2009;69:4894–903. 19487300 10.1158/0008-5472.CAN-08-3658 PMC3728826

[36] Gluzman M , Scott JG, Vladimirsky A. Optimizing adaptive cancer therapy: dynamic programming and evolutionary game theory. Proc R Soc B 1925;287:4–2020. 10.1098/rspb.2019.2454 PMC7211445 32315588

[37] Hansen E , Read AF. Modifying adaptive therapy to enhance competitive suppression. Cancers 2020;12:3556. 33260773 10.3390/cancers12123556 PMC7761372

[38] West J , You L, Zhang J et al. Towards multidrug adaptive therapy. Cancer Res 2020;80:1578–89. 31948939 10.1158/0008-5472.CAN-19-2669 PMC7307613

[39] Chandran D , Copeland WB, Sleight SC et al. Mathematical modeling and synthetic biology. Drug Discov Today: Dis Models 2008;5:299–309. 27840651 10.1016/j.ddmod.2009.07.002 PMC5102263

[40] Torres NV , Santos G. The (mathematical) modeling process in biosciences. Front Genet 2015;6:354. 26734063 10.3389/fgene.2015.00354 PMC4686688

[41] Winther RG. Mathematical modeling in biology: Philosophy and pragmatics. Front Plant Sci 2012;3:102. 22701118 10.3389/fpls.2012.00102 PMC3369192

[42] Zheng Y , Sriram G. Mathematical modeling: Bridging the gap between concept and realization in synthetic biology. J Biomed Biotechnol 2010;2010:1–16. 10.1155/2010/541609 PMC2878679 20589069

[43] Archetti M , Pienta KJ. Cooperation among cancer cells: applying game theory to cancer. Nat Rev Cancer 2018;19:110–7. 10.1038/s41568-018-0083-7 PMC8557269 30470829

[44] Brown JS. Why Darwin would have loved evolutionary game theory. Proceedings of the Royal Society B: Biological Sciences 2016;283. 10.1098/rspb.2016.0847 PMC5031650 27605503

[45] Lao Y , David J, Mirhadi A et al. Cancer treatment as a game: integrating evolutionary game theory into the optimal control of chemotherapy. Phys Biol 2012;9:65007–17. 10.1088/1478-3975/9/6/065007 PMC3653600 23197192

[46] McEvoy JW. Evolutionary game theory: lessons and limitations, a cancer perspective. British J Cancer 2009;101:2060–1. 10.1038/sj.bjc.6605444 PMC2795450 19920827

[47] Pacheco JM , Santos FC, Dingli D. The ecology of cancer from an evolutionary game theory perspective. Interface Focus 2014;4:8. 10.1098/rsfs.2014.0019 PMC4071510 25097748

[48] Weibull J. Evolutionary Game Theory. Cambridge, MA: MIT Press, 1987.

[49] Wolfl B , te Rietmole H, Salvioli M et al. The contribution of evolutionary game theory to under-´ standing and treating cancer. Dyn Games Appl 2022;12:313–42. 35601872 10.1007/s13235-021-00397-w PMC9117378

[50] Gavina MKA , Tahara T, Tainaka KI et al. Multi-species coexistence in LotkaVolterra competitive systems with crowding effects. Sci Rep 2018;8:1–8. 29352250 10.1038/s41598-017-19044-9 PMC5775205

[51] Morris SA , Pratt D. Analysis of the Lotka–Volterra competition equations as a technological substitution model. Technol Forecast Soc Change 2003;70:103–33.

[52] Apaloo J , Brown JS, Vincent TL. Evolutionary game theory: ESS, convergence stability, and NIS. Evol Ecol Res 2009;11:489–515.

[53] Bukkuri A , Brown JS. Evolutionary game theory: Darwinian dynamics and the G function approach. MDPI Games 2021;12:1–19.

[54] Christiansen FB. On conditions for evolutionary stability for a continuously varying character. Am Nat 1991;138:37–50.

[55] Smith JM , Price GR. The logic of animal conflict. Nature 1973;246:15–8.

[56] Rankin DJ , Bargum K, Kokko H. The tragedy of the commons in evolutionary biology. Trends Ecol Evol 2007;22:643–51. 17981363 10.1016/j.tree.2007.07.009

[57] Salvioli M , Dubbeldam J, Stankova K et al. Fisheries management as a stackelberg evolutionary game: finding an evolutionarily enlightened strategy. PLoS One 2021;16:e0245255. 33471815 10.1371/journal.pone.0245255 PMC7817040

[58] Bukkuri A , Adler FR. Viewing cancer through the lens of corruption: using behavioral ecology to understand cancer. Front Ecol Evolu 2021;0:442.

[59] Aguade-Gorgori´o G , Sole R. Adaptive dynamics of unstable cancer populations: the canoni-´ cal equation. Evol Appl 2018;11:1283–1292. 30151040 10.1111/eva.12625 PMC6099832

[60] Kisdi E , Geritz SAH. Adaptive dynamics: a framework to model evolution in the ecological theatre. J Math Biol 2010;61:165–169. 19777234 10.1007/s00285-009-0300-9

[61] Metz JJ , Geritz SH, Meszena G et al. Adaptive dynamics, a geometrical study of the consequences of nearly faithful reproduction. Stochastic and Spatial Structures of Dynamical Systems. Proceedings of the Royal Dutch Academy of Science (September), 1996.

[62] Vincent TL , Brown JS. Evolutionary Game Theory, Natural Selection, and Darwinian Dynamics. New York: Cambridge University Press, 2005.

[63] Basener WF , Sanford JC. The fundamental theorem of natural selection with mutations. J Math Biol 2018;76:1589–622. 29116373 10.1007/s00285-017-1190-x PMC5906570

[64] Frank SA , Slatkin M. Fisher’s fundamental theorem of natural selection. Trends Ecol Evol 1992;7:92–5. 21235964 10.1016/0169-5347(92)90248-A

[65] Lessard S. Fisher’s fundamental theorem of natural selection revisited. Theor Popul Biol 1997;52:119–36. 9356328 10.1006/tpbi.1997.1324

[66] Brannstrom A , Johansson J, von Festenberg N. The Hitchhiker’s guide to adaptive dynamics. Games 2013;4:304–28.

[67] Dieckmann U. Can adaptive dynamics invade? Trends Ecol Evol 1997;12:128–31. 21238006 10.1016/s0169-5347(97)01004-5

[68] Dionisio F , Gordo I. The tragedy of the commons, the public goods dilemma, and the meaning of rivalry and excludability in evolutionary biology. Evol Ecol Res 2006;8:321–332.

[69] Hardin G. The Tragedy of the Commons. Science 3859;162:2010. 5699198

[70] Yue E , Yang G, Yao Y et al. Targeting CA-125 Transcription by development of a conditionally replicative adenovirus for ovarian cancer treatment. Cancers 4265;13:8–2021. 34503075 10.3390/cancers13174265 PMC8428227

[71] Javanmardi S , Aghamaali M, Abolmaali S et al. miR-21, An oncogenic target miRNA for cancer therapy: Molecular mechanisms and recent advancements in chemo and radio-resistance. Curr Gene Ther 2017;16:375–89. 28042781 10.2174/1566523217666170102105119

[72] Javanmard SH , Vaseghi G, Ghasemi A et al. Therapeutic inhibition of microRNA-21 (miR-21) using lockednucleic acid (LNA)-anti-miR and its effects on the biological behaviors of melanoma cancer cells in preclinical studies. Cancer Cell Int 2020;20:384. 32788885 10.1186/s12935-020-01394-6 PMC7418194

[73] Lee TJ , Yoo JY, Shu D et al. RNA nanoparticle-based targeted therapy for glioblastoma through inhibition of oncogenic miR-21. Mol Ther 2017;25:1544–55. 28109960 10.1016/j.ymthe.2016.11.016 PMC5498802

[74] Antonia SJ , Mirza N, Fricke I et al. Combination of p53 cancer vaccine with chemotherapy in patients with extensive stage small cell lung cancer. Clin Cancer Res 2006;12:878–87. 16467102 10.1158/1078-0432.CCR-05-2013

[75] Cunningham JJ , Gatenby RA, Brown JS. Evolutionary dynamics in cancer therapy. Mol Pharm 2011;8:2094–100. 21815657 10.1021/mp2002279 PMC3250072

[76] Gatenby RA , Brown J, Vincent T. Lessons from applied ecology: cancer control using an evolutionary double bind. Cancer Res 2009;69:7499–502. 19752088 10.1158/0008-5472.CAN-09-1354

[77] Lee D-H , Choi S, Park Y et al. Mucin1 and Mucin16: therapeutic targets for cancer therapy. Pharmaceuticals 1053;14:10–2021. 10.3390/ph14101053 PMC8537522 34681277

[78] Li T , Wang J. Therapeutic effect of dual CAR-T targeting PDL1 and MUC16 antigens on ovarian cancer cells in mice. BMC Cancer 2020;20:678. 32689954 10.1186/s12885-020-07180-x PMC7372885

[79] Bukkuri A , Pienta K, Amend S et al. A life history model of the ecological and evolutionary dynamics of polyaneuploid cancer cells. Nature Scientific Reports 2022;12:1–25. 10.1038/s41598-022-18137-4 PMC9374668 35962062

[80] Bukkuri A , Pienta K, Amend S et al. Stochastic models of mendelian and reverse transcriptional inheritance in state-structured cancer populations. Nature Scientific Reports 2022;12:1–13. 10.1038/s41598-022-17456-w PMC9338039 35906318

[81] Turlings TCJ , Wackers, F. Recruitment of predators and parasitoids by herbivore-injured plants. Advances in Insect Chemical Ecology 2004;2:21–75.

[82] Adler FR. Plant signalling: the opportunities and dangers of chemical communication. Biol Lett 2011;7:161–2. 20880863 10.1098/rsbl.2010.0790 PMC3061173

